# Increased Incidence of Choroid Plexus Carcinoma Due to the Germline *TP53* R337H Mutation in Southern Brazil

**DOI:** 10.1371/journal.pone.0018015

**Published:** 2011-03-22

**Authors:** Gislaine Custodio, Guilherme R. Taques, Bonald C. Figueiredo, Elizabeth S. Gugelmin, Mirna M. Oliveira Figueiredo, Flora Watanabe, Roberto Pontarolo, Enzo Lalli, Luiz Fernando Bleggi Torres

**Affiliations:** 1 Pelé Pequeno Príncipe Research Institute, Curitiba, Paraná, Brazil; 2 Faculdades Pequeno Príncipe, Curitiba, Paraná, Brazil; 3 Pequeno Príncipe Hospital, Curitiba, Paraná, Brazil; 4 Department of Pharmaceutical Sciences, Federal University of Paraná, Curitiba, Paraná, Brazil; 5 Institut de Pharmacologie Moléculaire et Cellulaire, Valbonne, Alpes-Maritimes, France; 6 Université de Nice, Valbonne, Alpes-Maritimes, France; Virginia Commonwealth University, United States of America

## Abstract

**Background:**

Choroid plexus carcinomas (CPC) are rare tumors predominantly found in children. Given the high frequency of the germline R337H mutation in the *TP53* gene in southern Brazil, we have evaluated the frequency of the R337H mutation in families with CPC in children.

**Methodology/Principal Findings:**

The present series included 29 patients that were admitted to the same institution from 1992 to 2010, including 22 children with CPC (0.08–13.6 years of age at diagnosis) and 7 children with papilloma of the choroid plexus (Pp; 0.5–9.8 years of age). Surgical resection was possible in 28 children. Blood and/or tumor DNA was extracted and analyzed using PCR-RFLP and results were confirmed by sequencing 240 bp of the *TP53* exon 10. The patients, all parents, and some relatives submitted samples for blood DNA analysis. In addition, we have also examined the presence of the mutation in DNA from paraffin-embedded tumor samples to evaluate loss of heterozygosity. We found 63.3% (14/22) of the CPC patients positive for the germline R337H mutation; CPC samples were either heterozygous (n = 7), lost only the wild-type (n = 4), or only the R337H copy (n = 2). One CPC sample was not available. All Pp cases (7/7, 100%) were negative for R337H. Cure (>5 years survival free of disease) was observed in 18.1% of the CPC cases with the R337H mutation (2/11), 71.4% of the Pp (5/7), and 25% of CPC cases negative for the R337H mutation (2/8). Family history of cancer (with 2 or more cancer cases) was exclusively identified on the parental side segregating the R337H mutation, and 50% (7/14) of them were compatible with Li-Fraumeni-like syndrome.

**Significance:**

Our results show for the first time that the R337H *TP53* mutation is responsible for 63% of the CPC cases in children, suggesting a higher incidence of CPC in southern Brazil.

## Introduction

Choroid plexus carcinomas (CPC) are tumors originated from the epithelium lining the brain ventricles, and are typically found in children. CPC corresponds to 1–4% of all pediatric intracranial tumors [1] and are much less common than the benign intraventricular papillomas (Pp) [2]. CPC are very aggressive tumors, with a very unfavorable prognosis and typically appear in the first decade of life (38% of cases) [3]. The relatively small number of CPC cases in the literature, coupled with the lack of relevant epidemiological data and controversies about their clinical and pathological diagnosis makes it difficult to establish a standardized therapeutic approach in managing this condition [4], [5]. Considering the high mortality rate for CPC, it is very important to identify biologic markers that may be relevant to understanding risk stratification, tumor formation mechanism, and prognosis [6]. The most common mechanism involved in CPC formation is related to dysfunction in the tumor suppressor p53, more frequently found in families with Li-Fraumeni syndrome (LFS). It was recently reported that 50% of the patients with CPC were positive for germline *TP53* mutation [7]. These authors have concluded that children with CPC who have a p53 protein with low total structural variation and absence of *TP53* dysfunction have a favorable prognosis and can be successfully treated without radiation therapy.

Twenty four of the 29 cases included in the present study (24/29, 82.7%) were investigated in a previous histopathologic analysis claiming increased incidence of CPC in the state of Paraná, Brazil due to an unknown pathogen [8]. The present study was designed to address the question whether the increased number of CPC cases proposed in this previous study was related to the high frequency of the R337H germline mutation in southern Brazil [9], [10]. This mutation produces a 10–15 times higher adrenocortical tumor (ACT) incidence in southern Brazil than in the United States [11]. CPC is as rare as ACT in the United States, both occurring with an incidence of 0.3 per million of children younger than 15 years of age [2], [12], [13], [14].

## Materials and Methods

### Subjects

This study and the written consent form were approved by the Pequeno Príncipe Hospital Ethics Committee and the Federal IRB (CONEP, Brasilia, DF, Brazil). With approval and participation by their parents, samples (blood and/or tumor DNA) were collected from children admitted between 1992 and 2010 at Pequeno Príncipe Hospital (Curitiba, Brazil) and additional blood samples were obtained from some of their relatives after signing the consent form. A total of 22 cases of CPC from 15 boys and 7 girls, and 7 Pp from 4 girls and 3 boys were screened for the R337H mutation in the *TP53* gene. Histopathology was re-evaluated, and clinical and pedigree analyses were obtained. Involvement of one or both lateral ventricles was observed in all cases of CPC and Pp. The clinical manifestations were similar, and in most cases intracranial hypertension and/or hydrocephalus were observed. Surgical treatment was possible in 28 of the 29 patients and it was the only treatment for Pp. The protocol for chemotherapy (cisplatin and carboplatin) was similar for all patients with CPC. Five of the CPC patients were submitted to radiotherapy.

### Pedigree analysis and family history of cancer

One drop of blood was obtained (pricking of finger for subjects older than 3 years of age, or heel prick for children younger than 3 years). The blood drop was stored in DNA Cards (MGM, Curitiba, Brazil). Pedigrees and histories of cancer were obtained from each side of the family.

### PCR-RFLP assay

Two 2 mm punches of the DNA card from each sample were used 1–2 weeks later in the assay. Within this time period, it was sufficient to wash twice with washing solution (MGM Assessoria Biológica, Curitiba, Paraná) for 5 min at 37°C, and twice in autoclaved double distilled water (ddH_2_O) for 3 min. Punches were dried at 60°C for 15 min and stored at −20°C until used in PCR assays as previously described [9]. PCR was carried out in a 50 µl PCR buffer containing 2.5 U platinum Taq polymerase (Invitrogen, USA), 100 pmol of forward and reverse primers, 25 nmol each of deoxy-NTPs (Invitrogen) and 20–30 ng genomic DNA. The 447 base pair (bp) PCR product corresponding to a fragment of *TP53* exon 10 was generated using the following primers: 5′-CTG AGG CAC AAG AAT CAC-3′ (forward) and 5′-TCC TAT GGC TTT CCA ACC-3′ (reverse). Samples were denatured at 95°C for 45 seconds and amplified by 30 cycles (annealing at 62°C for 45 seconds and primer extension at 72°C for 45 seconds). The amplified product was digested with *Hha1*, yielding 293 bp and 154 bp fragments in 1.8% agarose gel, if the R337H point mutation was not present.

Genomic DNA from formalin-fixed and paraffin-embedded tumor tissue (FF-PET) samples were isolated using a protocol modified from Farrugia et al. [15]. Three 5-µM thick FF-PET sections from representative areas of tumor, avoiding necrosis and normal tissue (tissue block measuring 5 mm ×5 mm) were incubated in 1 mL autoclaved ddH2O at 70°C for 20 min, centrifuged for 3 min and the liquid with melted paraffin was discarded. This procedure was repeated twice. The tissues were incubated with proteinase K in lysis buffer overnight and the next steps were performed using a commercial kit for paraffin-embedded tissue (Qiagen). Part of the exon 10 of the *TP53* gene (240 kb) was amplified using polymerase chain reaction (PCR) using standard protocols. Approximately 30–50 ng of micro-dissected tumor genomic DNA was used in the PCR-RFLP assay. PCR-RFLP for DNA extracted from FF-PET was performed with some modifications: forward primer was replaced by 5′-TAA CTT GAA CCA TCT TTT AAC TC-3′, the annealing temperature was reduced to 61°C, electrophoresis was performed in 3% agarose gel for 70 min (10 min longer). The wild-type amplicon (240 bp) digested with *Hha1* yielded two fragments (85 and 160 bp). All cases identified with the R337H mutation were confirmed by sequencing the amplified 240 bp of exon 10.

### Statistical analysis

Progression-free survival rate for tumors positive for the germline mutation was compared with those negative for the mutation by using the Kaplan-Meier method. Chi-square test or Fisher's Exact Test were applied to compare categorical variables among the groups. The Kruskal-Wallis non-parametric test was used to compare continuous variables. A *P* value <0.05 was considered significant.

## Results

Fourteen children with CPC (14/22, 63.6%) were identified with the germline R337H *TP53* mutation (Table 1). This mutation was confirmed in blood DNA from at least one of the parents (n = 14) and in other members of the families (33 out of 115 tested individuals), ruling out a *de novo* mutation. Only 9 relatives of these children who had other types of cancer were tested and were found to be carriers of the R337H mutation (9/9). Loss of heterozygosity (LOH) was found in 46.1% (6/13) of the CPC cases, including 4 CPC with loss of the wild-type *TP53* and 2 with loss of the R337H haplotype, while the remaining available CPC (n = 7) were heterozygous (Table 1).

**Table 1:** Survival and history of cancer in the families of children with CPC carrying the R337H mutation.

Patient	Gender/Age(years)	Treatment	Outcome	Histopathology	Blood DNAR337H	Tumor DNAR337H	Family history of cancer	
							IPSI	CONTRA
							n	n
1	F/13.6	-	Deceased	**CPC**	R337H/Wt***	NA	4	0
2	M/1.8	CSR/Ct	Deceased	**CPC**	R337H/Wt	R337H/-	NA	1
3	M/2.5	CSR/Ct	Deceased	**CPC**	R337H/Wt	-/Wt	1	1
4	F/0.4	CSR/Ct	Deceased	**CPC**	R337H/Wt	-/Wt	4	0
5	M/11	CSR/Ct	Deceased	**CPC**	R337H/Wt	R337H/Wt	4	0
6	M/0.9	CSR/Ct	Alive	**CPC**	R337H/Wt	R337H/Wt	3	0
7	M/2.3	CSR/Ct	Deceased	**CPC**	R337H/Wt	R337H/Wt	7	NA
8	M/6.6	CSR/Ct	Deceased	**CPC**	R337H/Wt	R337H/Wt	4	1
9	M/2.9	CSR/Ct	Alive	**CPC**	R337H/Wt	R337H/Wt	8	0
10	M/1.2	CSR/Ct	Deceased	**CPC**	R337H/Wt	R337H/Wt	2	1
11	F/5.9	CSR/Ct	Alive*	**CPC**	R337H/Wt	R337H/-	0	NA
12	F/0.9	CSR/Ct	Deceased	**CPC**	R337H/Wt	R337H/Wt	3	NA
13	M/4	CSR/Ct	Alive*	**CPC**	R337H/Wt	R337H/-	5	0
14	M/0.08	CSR/Ct	Alive*	**CPC**	R337H/Wt	R337H/-	6	NA
Total				14	13	13	51	4
Survival rate**			2/11 (18.1%)					

CSR, Complete surgical removal; Ct, Chemotherapy; NA, Not available.

*<5 years free of disease;

**>5 years free of disease;

***DNA analysis estimated from their parents.

The number of cancer cases in each family was shown in the IPSI (ipsilateral) and CONTRA (contralateral) to the parental side segregating the R337H mutation.

**Figure 1:**
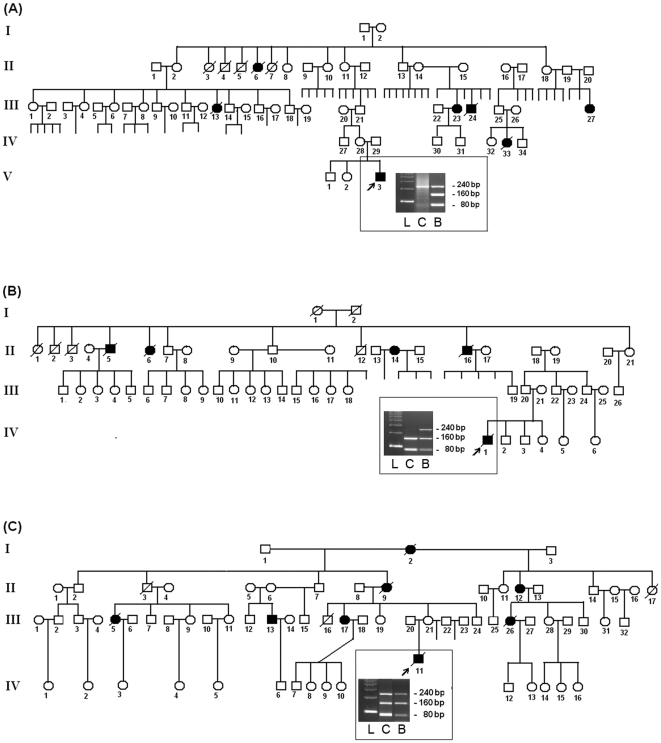
Typical pedigrees. The pedigrees of families with the germline *TP53* R337H mutation illustrate three different patterns of allele combinations shown in the digital photos of the gel, with loss of the wt allele in A (lane 1: DNA ladder; lane 2: PCR product from CPC DNA treated with *HhaI* and lane 3 with PCR product from the patient's blood DNA treated with *HhaI*), loss of the R337H copy in B (lane 1: DNA ladder; lane 2: PCR product from CPC DNA treated with *HhaI* and lane 3 with PCR product from the father's blood DNA treated with *HhaI*), and without any loss shown in C (lane 1: DNA ladder; lane 2: PCR product from CPC DNA treated with *HhaI* and lane 3 with PCR product from the patient's blood DNA treated with *HhaI*).

Figure 1 presents three pedigrees with an example for each situation: only one single 240 bp band depicting loss of the wild-type (Fig. 1A, shown in lane 2: PCR product from CPC DNA treated with *HhaI*, in contrast to the lane 3 profile with PCR product from the patient's blood DNA treated with *HhaI*), only two smaller bands, 80 bp and 160 bp, illustrating absence of the R337H copy (Fig. 1B, shown in lane 2: PCR product from patient's CPC DNA treated with *HhaI*, in contrast to the lane 3 profile with PCR product from his father's blood DNA treated with *HhaI*) and the heterozygous pattern with all three bands (Fig. 1C, shown in lane 2: PCR product from patient's CPC DNA treated with *HhaI*). All cases were confirmed by DNA sequencing of the patient or parental blood DNA, or in DNA from normal brain tissue surrounding CPC.

Age, gender, outcome, and family history of cancer are shown for CPC with the mutation (Table 1) and for CPC and Pp without the R337H mutation in the *TP53* gene (Table 2). Survival rate (>5 years free of disease) was not significantly different among patients with CPC and the mutation (2/11, 18.1%), CPC patients without the mutation (2/8, 25%) and patients with Pp without the mutation (5/7, 71.4%) probably due to the small number of cases. If we consider all CPC patients together (n = 22) and compare with Pp patients, the difference in survival is also not statistically significant.

**Table 2:** Survival and history of cancer in the families of children with CPC or Pp negative for the R337H mutation.

Patient	Gender/Age (years)	Treatment	Outcome	Histopathology	Blood DNAR337H	Tumor DNA R337H	Family history of cancer	
							Side 1*	Side 2
							n	n
15	F/9.2	CSR/Ct	Deceased	CPC	Neg	Neg	2	0
16	M/1.3	CSR/Ct	Deceased	CPC	Neg	Neg	2	1
17	M/4.9	CSR/Ct	Deceased	CPC	Neg	Neg	5	1
18	M/0.2	CSR/Ct	Deceased	CPC	Neg	Neg	2	0
19	F/3y	CSR/Ct	Deceased	CPC	Neg	Neg	0	0
20	M/0.3	CSR/Ct	Alive	CPC	Neg	Neg	0	0
21	M/0.7	CSR/Ct	Alive	CPC	Neg	Neg	1	0
22	F/1.7	CSR/Ct	Deceased	CPC	Neg	Neg	0	0
23	M/5.8	CSR	Alive	Pp	Neg	Neg	2	1
24	F/0.7	CSR	Deceased	Pp	Neg	Neg	2	1
25	F/0.8	CSR	Deceased	Pp	Neg	Neg	3	1
26	F/9.8	CSR	Alive	Pp	Neg	Neg	0	0
27	F/1y	CSR	Alive	Pp	Neg	Neg	0	NA
28	M/0.5	CSR	Alive	Pp	Neg	Neg	0	0
29	M/24	CSR	Alive	Pp	Neg	Neg	0	0
Total				CPC (n = 8)Pp (n = 7)	CPC (n = 8)Pp (n = 7)	CPC (n = 8)Pp (n = 7)	CPC (n = 12)Pp (n = 7)	CPC (n = 2)Pp (n = 3)
Survival rate**			CPC = 2/8 (25%)Pp = 4/6 (66.6%)					

CSR, Complete surgical removal; Ct, Chemotherapy;

*Parental side with higher number of cancer cases;

**>5 years free of disease.

The pedigrees of the 14 families with the germline mutation were obtained together with the history of cancer reported by both sides of the families; ipsilateral and contralateral to the parental side segregating the R337H mutation (Table 1). The total number of relatives with cancer in families with the germline R337H mutation (n = 51) was significantly higher than in the contralateral side of the family (n = 4) or in any side of the families of CPC patients without the mutation (n = 10) or with Pp patients (n = 7). The tumor types reported by all families revealed that the most common tumor types were: breast (n = 8, 12.1%), stomach (n = 6, 9.0%), prostate (n = 6, 9.0%) and ACT (n = 6, 9.0%) in families with CPC and the R337H mutation. None of the families presented classic LFS. However, LFS-like syndrome (LFL) as defined by Birch et al. [16], was identified in 7 of the 14 families with the R337H mutation (50%), and in 6 of the 14 families with the R337H mutation (42.8%) according to Tinat et al. [17]. None of the families contralateral to the parental side segregating the R337H mutation presented LFL. History of cancer in the families negative for the R337H mutation, presented LFL only in one family with CPC without the R337H mutation and none in families with Pp.

## Discussion

Although none of the families can be classified as LFS, LFL was clearly associated with the parental side segregating the R337H mutation. According to Aguzzi et al. [2], CPC affects more boys than girls (ratio 3:1), which is consistent with the present series of cases (15 boys and 7 girls). It was previously described that CPC represented 63.2% of all choroid plexus tumors in Curitiba, Brazil [8], in contrast to 8.1% (4) and 16.6% [2] in other series. This unusual higher proportion of CPC cases found in Curitiba was hypothesized to be related to an unknown pathogen [8].

This increased incidence of CPC can be accounted for by the presence of the *TP53* R337H germline mutation in the population of Paraná. It was previously described the germline R337H mutation in 35 of 36 children with adrenocortical cancer (ACC) using a GeneChip p53 probe array (Affymetrix, Santa Clara, CA) and sequence analysis of the *TP53* coding sequence and no other *TP*53 mutations were detected [9]. The hemizygous pattern with the R337H haplotype, after loss of the wild type *TP53* found in 4/14 CPC cases, reinforces the idea that the R337H protein is defective and may under certain metabolic conditions lose its protective role against cancer formation [18]. Remarkably, permanence of the wild-type allele in most CPC (9/13), including loss of the R337H copy (n = 2), challenge the general assumption that the tumor suppressor activity for *TP53* must be inactivated in the tumor. However, although the tumor tissue was carefully dissected, we cannot rule out the possibility of contamination with normal brain cells in the analyzed samples. It was previously found 100% of LOH (loss of the wild-type allele) in childhood adrenocortical cancer with the R337H germ-line mutation [9], [10], which is consistent with the classical two-hit mechanism of cancer proposed by Knudson [19]. In contrast, other studies have shown lower frequency of LOH (60–78%) in the same type of tumor with R337H [29], [30]. Furthermore, LOH was described in 3 cases of breast cancer, including loss of the mutant R337H and permanence of the wild-type *TP53* allele [20]. Conversely, in all families described in the present study the pedigrees show that the cancer cases are clearly associated with the R337H allele in one parental side, which reduces the remote possibility of a second mutation in the second copy of the *TP53* gene in the contralateral side of the families (negative for R337H), because none of them presented either LFS or LFL cancer history.

Functional analysis of the R337H p53 protein was previously performed using two cell types devoid of endogenous p53. Immortal murine BALB/c fibroblasts were used for promoter transactivation studies and human SaOS-2 osteosarcoma cells for colony reduction assays. The R337H mutant activated the reporter as efficiently as wild-type p53 did. Similarly, wild-type p53 and the R337H mutant were able to suppress colony growth of SaOS-2 cells [9]. One year later, DiGiammarino et al. [18], demonstrated that a pH-sensitive molecular defect of p53 (R337H) leading to diminished p53 tetramerization is the molecular basis for these cases of ACC in Brazilian children. They have also shown that supraphysiologic temperature contributes to loss of R337H function.

Considering that this peculiar *TP53* mutation is present in similar frequency in other states of southern Brazil, it is likely that the actual number of CPC in children caused by R337H per year is higher than the previously reported 0.3 per million incidence [1]. The population size in two states (Paraná and São Paulo), with 50 million inhabitants, including approximately 15 million children younger than 15 years of age, represents a serious public health concern. In a series of 31 families carrying the germline R337H mutation and 41 cases of ACT, none of them presented any case of CPC [10]. Most of the ACT in children reported to date in southern Brazil (85–95%) present the germline R337H mutation [11]. In addition, the total number of childhood ACT admitted to the main hospitals in the state of Paraná is much higher than the total number of children admitted with CPC. These observations are consistent with a lower R337H penetrance for CPC than for ACT in Brazilian children in these areas.

In a previous study, we had found LFL in only 23.3% of the families (7/30) presenting 40 cases of childhood ACT associated with 31 cases of other types of cancer; mainly breast (n = 5) and stomach (n = 5) [10]. In addition, in that previous study we described that the maximal number of cancer cases per family (excluding ACT) were 4 cases in two families and 2 cases in six families in the parental side segregating the R337H mutation. In contrast, in the present study the families carrying the R337H mutation are more frequently associated with a larger number of other tumor types, where 71.4% of the families with R337H (10/14) present 4 or more cancer cases and 50% (7/14) are LFL according to Birch et al. [16].

Does this association between CPC and other cancer cases reflect a different genetic profile than between ACT and other types of cancer? Interestingly, almost all patients (Table 2) without cases of cancer in their families are alive (*P*<0.05, Fisher's Exact Test), which is consistent with a lower malignancy of these tumors without the germline *TP53* R337H mutation (CPC and Pp).

In younger children, skull flexibility to accommodate an increased volume and the capacity of functional adaptation of the immature nervous system often allow CPC to reach large dimensions before being diagnosed, while older children tend to have typical signs of intracranial hypertension [8]. These authors have shown in their previous histopathologic analysis, and further confirmed by the re-evaluation of all present cases, that these tumors are characterized as either papillary or solid tumors composed of columnar or anaplastic epithelium, with signs of frank malignancy including marked nuclear pleomorphism, frequent mitosis, increased nucleus/cytoplasm ratio, high cell density, foci of necrosis and loss of demarcation between tumor cells and the surrounding brain tissue. The neuropathologic diagnosis can be sometimes difficult even with the help of immunohistochemistry and electron microscopy. In contrast, papillomas are benign tumors composed of proliferative cells similar to the normal choroid plexus. All histopathologic criteria proposed for malignancy [21] were found in all CPC of the present series: a) obvious invasion of adjacent neural tissue, b) loss of regular papillary architecture, c) marked nuclear pleomorphism, d) frequent mitoses increased with cell density, and e) presence of focal necrosis.

There may be some difficulty in the diagnosis of CPC due to their morphologic characteristics, similar to those of other tumors in routine microscopy, making it vital to find biologic markers to characterize these tumors. The differential diagnosis must be made with other neuroectodermal tumors, such as ependymomas, metastatic papillary carcinomas (uncommon in children), embryonal carcinomas, anaplastic meningiomas, glioblastomas, and malignant pituitary adenomas, among others [21], [22]. Some immunohistochemical markers are proposed, including glial acid fibrillary protein (22–85%), S-100 protein (40–94%); vimentin (16–88%), epithelial antigen membrane (69-71%), neuron-specific enolase (20–100%), prealbumin (67–100%), carcinoembryonic antigen (0–50%) and cytokeratin (83–100%) [23].

The mechanisms involved in CPC formation are not clear and the reported causes are scarce. Previous reports suggested that inactivation of hSNF5/INI-1 might play a special role in CPC development [6]. Furthermore, it was recently reported that loss of p53 function seems to be the most prevalent cause [7]. These authors have found germline *TP53* mutation in 50% of the cases (32/64). They also have reported that two sequence variants known to confer *TP53* dysfunction—*TP53* codon72 and MDM2 SNP309—coexist in the majority of *TP53* wild-type CPC (92%) and not in *TP53*-mutated CPC, which suggests a complementary mechanism of *TP53* dysfunction in the absence of a *TP53* mutation.

CPC has also been associated with Simian Virus 40 (SV40), a primate polyomavirus potentially oncogenic in rodents, suggested by the identification of DNA sequences in 50% of the CPC cases in children [24]. Interestingly, DNA tumor virus proteins, including SV40, HPV E6, and the adenovirus protein E1B-55k inactivate p53 via high-affinity protein-protein interaction [25]. In addition, another epigenetic mechanism of p53 inactivation mediated by a viral protein that silences p53-activated transcription, irrespective of p53 phosphorylation and stabilization, was recently reported [26]. These authors have demonstrated that the adenovirus protein E4-ORF3 may target p53 promoters as part of a general anti-viral transcriptional silencing program, without co-localizing with p53. These viral proteins are important candidates to be further investigated in all cases of CPC and Pp.

The treatment of choice for CPC is surgical resection of the tumor block, a potentially curative procedure that may confer up to 50% survival in the series with the best results [27]. Radiotherapy has been recommended to reduce the tumor before surgery and to control it in cases of locally-invasive disease, disseminated or recurrent [1]. However, only five CPC patients of the present series were submitted to radiotherapy, and only three of them survived (two negative and one positive for the R337H mutation). Chemotherapy is presented as a valuable instrument to be associated with surgical treatment. There is, however, no consensus on the chemotherapy of choice because of the difficulty in drawing up protocols in view of the rarity of the tumor [28].

It was shown that patients with CPC presenting low tumor total structural variation (TSV) and absence of *TP53* dysfunction have a favorable prognosis and can be successfully treated without radiation therapy [7]. In contrast, these authors have shown that high TSV was associated with significant risk of progression and that five-year survival rates for patients with *TP53*-immunopositive and immunonegative CPCs were 0% and 82%, respectively. They also have shown that 14 of 16 patients with *TP53* wild-type CPC are alive without having received radiation therapy. Survival rate (>5 years free of disease) in our present series was 18.1% (2/11) for R337H positive CPC (Table 1) and 22% for R337H-negative CPC (Table 2). This lower survival rate in R337H-negative CPC, in contrast to the Canadian series (82%), may be due to differences in treatment protocols, or delay in tumor diagnosis. Patients with germline *TP53* mutations seem to be at a higher risk of secondary radio-induced malignancies due to the risk of inducing new mutations in the wild-copy of the gene [31], [32]. Further studies are necessary to evaluate the risk/benefit ratio of postoperative radiotherapy in long-term CPC survivors carrying the germline R337H mutation.

In conclusion, the R337H *TP53* mutation may be responsible for an increased CPC incidence in Curitiba and probably in other regions of southern Brazil, where the mutation was already documented to be very frequent in ACT (around 85–95% in the states of São Paulo and Paraná) [9], [11], [29]. Further studies of *TP53* mutations are necessary to understand their contribution to CPC formation and their participation in CPC prognosis. It will be necessary to continue identifying relatives positive for the R337H mutation in order to provide genetic counseling and orientation for precocious cancer diagnosis.
